# Survival benefit of transarterial chemoembolization in patients with metastatic hepatocellular carcinoma: a single center experience

**DOI:** 10.1186/s12876-017-0656-z

**Published:** 2017-08-10

**Authors:** Dominik Bettinger, Renan Spode, Nicolas Glaser, Nico Buettner, Tobias Boettler, Christoph Neumann-Haefelin, Thomas Baptist Brunner, Eleni Gkika, Lars Maruschke, Robert Thimme, Michael Schultheiss

**Affiliations:** 1 Department of Medicine II, Medical Center University of Freiburg, Faculty of Medicine, University of Freiburg, Hugstetter Str. 55, D-79106 Freiburg, Germany; 2 Berta-Ottenstein-Programme, Faculty of Medicine, University of Freiburg, Freiburg, Germany; 3 Department of Radiation Oncology, Medical Center University of Freiburg,Faculty of Medicine, University of Freiburg, Robert-Koch-Str. 3, D-79106 Freiburg, Germany; 4 Department of Radiology, Medical Center University of Freiburg, Faculty of Medicine, University of Freiburg, Hugstetter Str. 55, D-79106 Freiburg, Germany

**Keywords:** Hepatocellular carcinoma, Metastases, Sorafenib, Transarterial chemoembolization, Prognosis

## Abstract

**Background:**

As prognosis of patients with metastatic hepatocellular carcinoma (HCC) is mainly determined by intrahepatic HCC progression, local treatment with TACE may result in improved OS, although it is not recommended. The purpose of this study was to analyze retrospectively the efficacy of TACE and its impact on OS in patients with metastatic hepatocellular carcinoma (HCC).

**Methods:**

Two hundred and fifteen patients with metastatic HCC who were treated at our Liver Center between 2003 and 2014 were included in this retrospective analysis. Medical records, laboratory parameters and imaging studies were analyzed. Treatment of metastatic HCC and OS were assessed

**Results:**

One hundred and two patients (47.4%) did not receive any HCC specific treatment while 48 patients (22.3%) were treated with sorafenib, 42 patients (19.5%) with TACE and 23 patients (10.7%) received treatment with TACE and sorafenib in combination. Survival analyses and Cox regression models revealed that TACE and a combination therapy of TACE and sorafenib were significant prognostic factors in metastatic HCC. However, further analyses revealed that there was no additional prognostic effect of adding sorafenib to TACE treatment in this patient cohort.

**Conclusions:**

In metastatic HCC, treatment of intrahepatic tumor by TACE may be associated with improved survival. These results support the prognostic importance of treating intrahepatic HCC even in patients with metastatic disease. Therefore, we suggest evaluating the technical feasibility of TACE in all metastatic patients.

**Electronic supplementary material:**

The online version of this article (doi:10.1186/s12876-017-0656-z) contains supplementary material, which is available to authorized users.

## Background

Hepatocellular carcinoma (HCC) is the fifth most common cancer worldwide and its incidence is increasing due to the high incidence of non-alcoholic steatohepatitis (NASH) in the Western world [1–3]. Moreover, HCC is the third leading cause of cancer-related deaths worldwide [4]. Detection of HCC in surveillance programs has markedly improved, but patients with HCC are often diagnosed in advanced stages with the presence of vascular invasion or with extrahepatic tumor spread that is present in 15-42% of patients [5–10]. Prognosis in these patients is limited as there are no curative treatment options available.

Treatment of advanced HCC is determined according to the Barcelona Clinic Liver Cancer (BCLC) classification. Patients with advanced HCC, defined as presence of portal vein invasion or extrahepatic spread, are classified as BCLC stage C and if liver function or the performance status deteriorates, they are staged as BCLC D. In patients with BCLC stage C with and without metastases, sorafenib is the treatment of choice although it only leads to a modest improvement of overall survival (OS) compared to treatment with best supportive care [11, 12]. Previous studies have shown that prognosis of patients with metastatic HCC is mainly determined by intrahepatic HCC, hepatic failure due to progression of intrahepatic tumor disease or progression of the underlying liver disease rather than by extrahepatic metastases [13, 14]. These results provide the rationale for treatment of intrahepatic HCC in order to preserve liver function and argue against systemic treatment. In the last years, several studies revealed that transarterial chemoembolization (TACE), the recommended treatment in patients with intermediate HCC, can also be safely and effectively performed in patients with advanced HCC defined by vascular invasion or extrahepatic spread [15]. As patients with advanced HCC are a very heterogeneous group with vascular invasion, extrahepatic metastases or both, it is not clear which subgroup will benefit most from an intrahepatic treatment approach using TACE [10, 16]. The aim of this retrospective study was to assess the efficacy of TACE and its impact on OS in patients with metastatic hepatocellular carcinoma (HCC).

## Methods

### Selection of patients

Between January 2003 and January 2013 1030 patients who presented with newly diagnosed HCC at our Liver Center were included in an HCC database. 215 of these patients (20.9%) presented with metastatic HCC and were included in these analyses. Patients with history of malignancies other than HCC within the last 5 years were excluded from the analyses to ensure that present metastases were linked to HCC. Further, patients who have been treated with systemic chemotherapy or with radiation therapy in clinical studies were not included in the analyses. Demographic data including etiology of liver disease, blood count, liver function test, the international normalized ratio (INR), alpha-fetoprotein (AFP) and tumor characteristics were collected from the electronical medical records and included in the database retrospectively.

### Definitions and methods

HCC was diagnosed according to current guidelines by histopathology or computerized tomography (CT) scan or dynamic contrast-enhanced magnetic resonance imaging (MRI) showing the typical hallmark of HCC imaging (hypervascularity in the arterial phase with washout in the portal venous or delayed phases) [17, 18]. The number of focal hepatic lesions, the maximum tumor diameter and portal vein thrombosis and its extent were detected during contrast enhancement. The numbers of intrahepatic lesions are summarized in oligonodular (one or two intrahepatic lesions) and in multifocal HCC (three or more lesions or diffuse HCC growth pattern). HCC was staged according to the Barcelona Clinic Liver Cancer (BCLC) classification.

The site of metastasis was determined by CT or MRI scan of the chest and abdomen. In the case that suspected bone metastases were not sufficiently classified in the mentioned imaging modalities, bone scintigraphy was performed for confirmation.

Liver function was assessed using the recently developed ALBI score [19]. The ALBI score was calculated using the following equation: linear predictor = (log_10_ bilirubin μmol/l × 0.66) + (albumin g/l × −0.085). Bilirubin was recorded in mg/dl and albumin in g/dl, but for the calculation of the linear predictor of the ALBI score, these parameters were transformed in the corresponding units (bilirubin in μmol/l and albumin in g/l). The linear predictor of the ALBI score was categorized in three prognostic groups as published by Johnson et al. [19]: grade 1 (less than −2.60), grade 2 (between −2.60 and −1.39) and grade 3 (above −1.39) with a higher ALBI score being associated with an impaired liver function.

### TACE procedure

All HCC patients were discussed interdisciplinary in a review board with hepatologists, interventional radiologists, surgeons, nuclear medicine physicians and radiotherapists. As TACE is not recommended as the treatment of choice for patients with metastatic HCC, the decision to perform TACE was made on an individual basis in each patient. Clinical data, such as liver function and the ECOG performance score, as well as tumor characteristics including portal vein thrombosis and the hepatic vascular architecture were reviewed. If TACE was technically feasible and the patient presented in good performance status and preserved liver function, TACE was performed using a selective or super-selective approach. Intra-arterial infusion of the chemotherapeutic agent and lipiodol was performed after having localized the target lesion. Epirubicin or mitomycin (doses of max. 100 mg) were used as chemotherapeutic agents in our group. The chemotherapeutic agent was not defined in the study protocol. The lipiodol infusion was stopped when intra-arterial stasis was observed in the angiographic control. Further, gelatin sponge particles or PVA particles were used for embolization. The extent of embolization was selected individually. On the total number of 65 patients treated with any type of TACE, 11 cases received drug-eluting beads TACE (DEB-TACE; 16.9%) and 54 patients were treated with conventional TACE (cTACE; 83.1%). A mean number of 1.7 TACE sessions were performed on demand.

### Sorafenib treatment

Decision for sorafenib treatment was also made in an interdisciplinary review board. In total, 71 patients were treated with sorafenib. The full dose of 800 mg sorafenib per day was administered only in 16 of the 71 patients (22.5%) receiving sorafenib treatment. In the remaining 55 of the 71 patients (77.5%), sorafenib was started at a dose of 400 mg per day. Only in 58% of these patients, the dose of sorafenib could be increased to 800 mg per day due to side effects. The median time of sorafenib application was 59 (2 – 690) days.

### Statistical analyses

The present study was a retrospective observational study. All patients were followed-up until death or last contact. At the end of the observation 194 of the 215 analyzed patients (90.2%) had died. The primary endpoints were the administered treatment modalities as well as OS stratified according to the individual therapy modalities. OS was calculated from the day of detection of metastases. The cut-off point for survival data was 25th of February 2017.

Continuous variables are expressed as median with the minimum and maximum whereas categorial variables are reported as frequencies and percentages unless stated otherwise.

For continuous variables, differences were determined using Wilcoxon-Mann-Whitney and Kruskal-Wallis tests as there was no Gaussian distribution of the data confirmed by the Kolmogorov-Smirnov test. χ^2^ tests or Fisher’s Exact tests were used for categorial variables. For sub-analyses of statistically significant tests, the Bonferroni correction was applied. *P* values <0.05 were considered being significant.

Overall survival was calculated using Kaplan Meier analyses with death being recorded as event. Differences in survival were assessed using logRank tests. To analyze prognostic factors univariate Cox regression models were performed. Age as a continuous variable, gender, ECOG performance score, intrahepatic tumor expansion (oligonodular vs. multifocal), BCLC (stage C vs. D), treatment modalities, the ALBI score, segmental portal vein thrombosis and etiology of liver disease (stratified in viral and non-viral liver disease), tumor size expressed as the large tumor diameter and AFP as a categorial variable with a cut-off of 400 ng/ml were included in the regression model. The ALBI score was used for the assessment of liver function as the Child score incoporates investigator-dependent variables such as hepatic encephalopathy and ascites which might be biased by the retrospective design of the study. Laboratory parameters representing liver function were not included in the models to avoid collinearity with regard to the ALBI score. After univariate analyses of possible predictive factors, multivariate Cox regression model was established using the forward selection method. A limit of *p* < 0.05 for candiate variables to enter the stepwise Cox model was used.

Subgroup analyses were performed in patients who were treated before and after introduction of sorafenib in daily clinical practice in 2007.

Statistical analyses were performed with SPSS (version 24.0, IBM, New York, USA) and GraphPad Prism (version 6, GraphPad Software, San Diego, CA, USA).

## Results

### Patient characteristics

Demographic and clinical characteristics of the 215 enrolled patients at the time of study inclusion are summarized in Table 1. The median age was 69 (33 – 87) years. 68.4% of the patients presented with non-viral liver disease while 31.6% of the patients had viral liver disease. 60.3% of these patients had chronic hepatitis B virus (HBV) infection and 39.7% were diagnosed with chronic hepatitis C virus (HCV) infection. In total, 35 patients of the included 215 patients (16.3%) had cyroptogenic liver cirrhosis. At the time of HCC diagnosis, the underlying etiology of the liver cirrhosis was not clearly assessable in these patients. Probably, some of them may have had previous non-alcoholic fatty liver disease and due to progression of liver cirrhosis they developed sarcopenia so that the typical features of non-alcoholic fatty liver disease were not present anymore. All patients had typical imaging features of liver cirrhosis in ultrasound examination or in CT or MRI imaging. Assessing liver function with the ALBI score, 24,7% were classified as ALBI 1, 56.7% as ALBI 2 and 18.6% as ALBI 3. In comparison 67.9% of the included patients were classified Child A, 26.5% Child B and 5.6% Child C.

**Table 1:** Baseline characteristics of study patients

	All patients	No treatment	Sorafenib	TACE	TACE and sorafenib	*p* value^#^
Parameter	*n* = 215	*n* = 102	*n* = 48	*n* = 42	*n* = 23	
Epidemiology						
Gender, m/f (%)	185/30 (86.0/14.0)	85/17 (83.3/16.7)	42/6 (87.5/12.5)	36/6 (85.7/14.3)	22/1 (95.7/4.3)	0.480
Age, median (min.- max.)	69 (33 – 87)	70 (41 -87)	64 (33 – 85)	68 (46 – 85)	70 (50 -87)	0.150
ECOG						0.163
0	149 (69.3)	65 (63.7)	35 (72.9)	29 (69.1)	20 (87.0)	0.164
1	31 (14.4)	14 (13.7)	6 (12.5)	8 (19.0)	3 (13.0)	0.844
2	35 (16.3)	23 (22.6)	7 (14.6)	5 (11.9)	0	0.042
Etiology of liver disease						
Viral (%)	68 (31.6)	29 (28.4)	16 (33.3)	16 (38.1)	7 (30.4)	0.718
HBV (%)^|^	41 (60.3)	18 (62.1)	7 (43.8)	12 (75.0)	4 (57.1)	
HCV (%)^|^	27 (39.7)	11 (37.9)	9 (56.2)	4 (25.0)	3 (42.9)	
Non-viral (%)	147 (68.4)	73 (71.6)	32 (66.7)	26 (61.9)	16 (69.6)	0.658
Alcohol (%)^|^	82 (55.7)	42 (57.5)	18 (56.3)	14 (53.8)	8 (50.0)	
NAFLD (%)^|^	21 (14.4)	12 (16.4)	1 (3.1)	6 (23.1)	2 (12.4)	
cryptogenic (%)^|^	35 (23.8)	16 (21.9)	9 (28.1)	6 (23.1)	4 (25.0)	
hemochromatosis (%)^|^	8 (5.4)	3 (4.1)	4 (12.5)	-	1 (6.3)	
autoimmune (%)^|^	1 (0,7)	-	-	-	1 (6.3)	
Child Score						
Score, median	5 (5-14)	6 (5-14)	5 (5-9)	5 (5-9)	5 (5-9)	0.001
(min.-max.)					18 (78.3)	0.003
Child A	146 (67.9)	57 (55.8)	38 (79.2)	33 (78.6)	5 (21.7)	0.005
Child B	57 (26.5)	33 (32.4)	10 (20.8)	9 (21.4)	0	0.340
Child C	12 (5.6)	12 (11.8)	0	0		0.004
ALBI score^##^median (min.- max.)	−2.11 (−4.69 – −0.32)	−1.79 (− 3.25 – −0.32)	−2.36 (−3.36 – −0.85)	−2.19 (−4.69 – −0.76)	−2.23 (−3.32- - 1.13)	<0.001
ALBI grade^##^(%)						<0.001
ALBI 1	53 (24.7)	16 (15.7)	17 (35.4)	11 (26.2)	9 (39.1)	0.018
ALBI 2	122 (56.7)	54 (52.9)	29 (60.4)	28 (66.7)	11 (47.9)	0.355
ALBI 3	40 (18.6)	32 (31.4)	2 (4.2)	3 (7.1)	3 (13.0)	<0.001
Tumor characteristics						
Intrahepatic HCC: oligonodular vs. multifocal (%)	51 / 149 (26.6/73.4)	22 /80 (21.6/78.4)	8 /40 (16.7/83.3)	17 / 25 (40.5/59.5)	7/16 (30.4/69.6)	0.071
Segmental portal vein thrombosis (%)	36 (17.4)	19 (18.6)	5 (10.4)	7 (16.7)	5 (21.7)	0.611
BCLC (%)						<0.001
C	183 (85.1)	74 (72.5)	44 (91.7)	42 (100)	23 (100)	
D	32 (14.9)	28 (27.5)	4 (8.3)	0	0	
Largest tumor diameter [cm], median (min.-max.)	5.2 (1 – 18)	5.8 (1 – 17	4.4 (1 – 17)	5.3 (1- 17)	7 (2- 18)	0.308
Largest tumor size >7 cm (%)	66 (30.7)	34 (33.3)	9 (18.8)	12 (28.6)	11 (47.8)	0.078
Location of metastases						0.006
Lung	65 (30.1)	38 (37.3)	11 (22.9)	9 (21.4)	7 (30.4)	0.012
Bone	24 (11.2)	11 (10.8)	5 (10.4)	3 (7.1)	5 (21.7)	0.012
Lymph nodes	42 (19.5)	14 (13.7)	8 (16.7)	17 (40.5)	3 (13.1)	0.012
Peritoneum	12 (5.6)	5 (4.9)	3 (6.3)	3 (7.1)	1 (4.4)	0.718
Adrenal gland	4 (1.9)	3 (2.9)	0	1 (2.4)	0	0.425
Others	18 (8.4)	10 (9.8)	2 (4.2)	6 (14.3)	0	0.416
Multiple sites^*^	50 (23.3)	21 (20.6)	19 (39.5)	3 (7.2)	7 (30.4)	0.002
Laboratory analyses Median (min.-max.)						
White blood count [10^6^/μl]	6. 7 (2.1 – 34.4)	6,4 (2.1 – 28.0)	6.8 (2.6 – 23.0)	6.6 (2.4 – 34.4)	6.8 (2.6 – 14.4)	0.689
Platelets [10^6^/μl]	157 (9 -702)	150 (9 -702)	201 (29 – 363)	145 (56 – 480)	161 (51 -401)	0.400
Hemoglobin [g/dl]	12.3 (6.8 – 19.1)	11.9 (6.8 – 16.4)	12.9 (7.8 – 19.1)	13.1 (8.9 – 17.3)	12.6 (7.8 -14.9)	0.002
INR	1.13 (0.7 – 10.0)	1.17 (0.7 – 10.0)	1.12 (0.8 – 2.6)	1.10 (0.8 – 3.4)	1.01 (0.8 – 3.2)	0.078
Creatinine [mg/dl]	0.9 (0.4 – 7.4)	0.9 (0.4 – 5.5)	0.9 (0.5 – 7.4)	0.9 (0.4 – 2.1)	0.9 (0.6 – 1.3)	0.123
AST [U/l]	75 (12 – 674)	96 (18 – 674)	75 (25 – 450)	56 (12 – 252)	65 (20 -472)	0.002
ALT [U/l]	47 (8 - 640)	48 (13 -640)	49 (16 – 205)	45 (8 – 234)	46 (15 – 253)	0.555
Bilirubin [mg/dl]	1.0 (0.3 – 30.9)	1.5 (0.3 -30.9)	0.9 (0.3 – 6.8)	0.9 (0.4 – 14.9)	0.8 (0.4 – 2.5)	<0.001
Albumin [g/dl]	3.5 (2.0 – 6.4)	3.3 2.0 – 4.7)	3.7 (2.4 – 4.6)	3.5 (2.5 – 6.4)	3.7 (2.4 – 4.6)	<0.001
AFP [ng/ml]	222.0 (0.6 – 502,900.0^**^)	303.1 1.1 – 502,900.0)	174.6 (1.1 – 60,500.0^**^)	71.4 (0.6 – 60,500.0)	174.0 (1.5 – 60,500.0)	0.098
AFP > 400 ng/ml (%)	93 (43.3)	49 (48.0)	20 (41.7)	16 (38.1)	8 (34.8)	0.557

# *p* values are referred to group comparisons between the different therapies

| relative frequencies are referred either to viral or non-viral etiology

## a *higher* ALBI score is associated with *impaired* liver function. ALBI grade 1 represents good liver function while ALBI 3 shows worse liver function

*All patients with multiple metastases had pulmonary metastases

^**^ 502,900 ng/ml and 60,500 ng/ml were the highest values which could have been measured with the AFP assay. As the assay has changed the highest measurable values has also changed

All patients had advanced HCC as classified according to the BCLC classification. One hundred and eighty three patients (85.1%) were in BCLC C and 32 patients (14.9%) were in BCLC D. 73.4% of the patients had multifocal intrahepatic HCC and 30.7% displayed HCC larger than 7 cm. Intrahepatic segmental portal vein thrombosis was detected in 17.4%. None of the patients had extrahepatic portal vein thrombosis.

30.2% of the patients were diagnosed with pulmonary metastases and in 23.3% metastases at multiple sites (all with pulmonary metastases) were found. Lymph node metastases, bone, peritoneal and adrenal gland metastases were found in 19.5%, 11.2%, 5.6% and 1.9%, respectively.

### Treatment of patients with metastatic HCC

In our cohort,102 patients (47.4%) with metastatic HCC did not receive any HCC specific therapy. Forty-eight patients (22.3%) were treated with sorafenib. In 42 patients (19.5%) TACE was performed as an individual treatment approach as outlined in the methods section. In 23 patients (10.7%), TACE was performed in combination with ongoing sorafenib treatment. In 17 patients (73.9%) sorafenib was started 5.5 ± 2.0 days after TACE and in 6 patients (26.1%) sorafenib was ongoing when TACE was additionally performed.

**Fig. 1:**
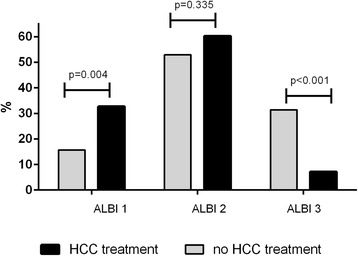
Liver function assessed by the ALBI score in patients with no HCC specific treatment compared to patients with different treatment approaches. Patients who did not receive any HCC specific treatment had impaired liver function as shown by a higher ALBI grade. * The group “HCC treatment” includes all patients who received either sorafenib, TACE or a combination of both

Compared to patients who received sorafenib, TACE or a combination of both, patientswho did not receive any HCC specific therapy had impaired liver function as indicated by a higher ALBI score (Table 1, Fig. 1). The ALBI score did not differ significantly between patients who had been treated with TACE, sorafenib or a combination of both (*p* = 0.579).

Patients with multiple extrahepatic metastases were also treated less commonly compared to patients with solitary metastases (*p* = 0.002, Table 1). In this subgroup sorafenib was significantly more often used than TACE (39.6% vs. 7.1%, *p* < 0.001, Table 1). The site of the metastasis did not influence the treatment approach.

Patients with BCLC D did not receive any HCC specific treatmentexcept from four patients who were treated with sorafenib. The decision to treat these patients against current guideline recommendations was based on an interdisciplinary discussion.

The intrahepatic growth pattern (oligonodular vs. multifocal, *p* = 0.071) and the size of the largest tumor diameter (*p* = 0.308) did not differ between the treatment approaches.

We included patients who had been treated between 2003 and 2013. Before 2007 sorafenib had not been introduced in daily clinical practice for treatment of HCC. Therefore, we divided the cohort in two sub-groups (treatment before [*n* = 83] and after 2007 [*n* = 132]). Before 2007, 64 of 83 patients (77.1%) did not receive any HCC-specific treatment while 19 of 83 patients (22.9%) were treated with TACE. After sorafenib had been introduced in daily clinical practice, 48 of 132 patients (36.4%) were treated with sorafenib, 23 patients (17.4%) received TACE, and another 23 patients (17.4%) were treated with a combination of TACE and sorafenib. Thirty-eight of 132 patients (28.8%) received no specific HCC treatment. In both groups, impaired liver function was the main reason for withholding HCC-specific treatment (Additional file 1: Figure S1). Further, there were no differences concerning portal vein invasion or thrombosis, intrahepatic tumor expansion or site and number of extrahepatic metastases between the different treatment approaches in both sub-groups.

### Influence of HCC treatment on overall survival and prognostic factors

**Fig. 2:**
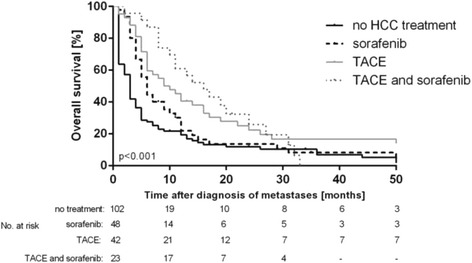
Patients who did not receive any HCC specific treatment had worst median OS of 3 months compared to patients who were treated with sorafenib (6 months, *p* = 0.009), with TACE (9 months, *p* < 0.001) and to those treated with a combination of TACE and sorafenib (16 months, *p* = 0.002)

Median OS of all included patients was 5.0 [4.0 – 6.0] months. Patients who did not receive any HCC specific treatment had a median OS of 3 months [95% CI: 2.01 – 3.95] compared to patients who were treated with sorafenib (6 months [95% CI: 4.67 – 7.33], *p* = 0.009), with TACE (9 months [95% CI: 4.46 – 13.54], *p* < 0.001) and to those who were treated with a combination of TACE and sorafenib (16 months [95% CI: 9.94 – 22.10], *p* = 0.002) (Fig. 2). These results were confirmed in a multivariate Cox regression model showing that TACE (HR 0.50 [0.33 – 0.76], *p* = 0.001) and a combination of TACE and sorafenib (HR 0.39 [0.23-0.66], *p* < 0.001) were significant independent prognostic factors in patients with metastatic HCC (Table 2). These results were confirmed in the sub-groups (treatment before and after 2007).

**Table 2:** Prognostic factors in patients with metastatic HCC

Parameters	Univariate			Multivariate		
	HR^1^	95% CI^2^	p	HR	95% CI	p
Age	0.99	0.98 - 1.01	0.745			
gender (male vs. female)	1.43	0.96 - 2.14	0.082			
Intrahepatic tumor expansion (multifocal vs. oligonodular)	1.74	1.23 - 2.47	0.002	1.63	1.11 - 2.42	0.014
Tumor size (cm)	1.00	0.97 - 1.04	0.889			
ECOG			0.127			
0	1					
1	1.04	0.69-1.58				
2	1.50	1.01-2.22				
Treatment			<0.001			0.007
No therapy	1			1		
Sorafenib	0.62	0.43 – 0.91	0.013	0.86	0.56 – 2.26	0.475
TACE	0.45	0.30 – 0.66	<0.001	0.54	0.35 – 0.84	0.006
TACE and sorafenib	0.41	0.25 – 0.68	<0.001	0.48	0.28 – 0.81	0.006
ALBI			<0.001			<0.001
ALBI 1	1			1		
ALBI 2	1.63	1.15 - 2.31	0.006	1.56	1.10 - 2.30	0.026
ALBI 3	3.33	2.15 - 5.15	<0.001	3.42	2.09 - 5.61	<0.001
viral vs. non-viral etiology	0.89	0.65 - 1.20	0.432			
Segmental portal vein thrombosis	1.12	0.97 - 1.29	0.128			
AFP > 400 ng/ml	1.51	1.13 - 2.01	0.005	1.50	1.10 - 1.92	0.010
BCLC (C vs. D)	5.15	3.31 - 8.02	<0.001	2.45	1.42 – 4.29	0.001
multiple metastases	0.96	0.69 - 1.33	0.797			

TACE is an independent prognostic factor in patients with metastatic HCC. Moreover, liver function represented by the ALBI score is also a strong prognostic factor indicating the importance of a preserved liver function for OS in these patients

*Abbreviations:* ^*1*^ *HR* hazard ratio, ^*2*^ *95% CI* 95% confidence interval

In order to analyze the additional effect of sorafenib on OS in metastatic patients treated with TACE, a sub-group analysis including only patients who have either been treated with TACE (*n* = 42) or with a combination of TACE and sorafenib (*n* = 23) was performed. Multivariate Cox regression analysis revealed that additional treatment with sorafenib was not an independent prognostic factor. Intrahepatic tumor burden (HR 1.86 [0.99 – 3.47], *p* = 0.053) was an independent negative prognostic factor in these patients (Table 3).

**Table 3:** Prognostic factors in patients with metastatic HCC treated with TACE or TACE and sorafenib in combination

Parameters	Univariate			Multivariate		
	HR^1^	95% CI^2^	p	HR	95% CI	p
Age	1.01	0.98 – 1.04	0.725			
gender (male vs. female)	2.27	1.00 – 5.11	0.048	2.39	0.99 – 5.76	0.052
Intrahepatic tumor expansion (multifocal vs. oligododular)	1.95	1.07 – 3.55	0.028	1.86	0.99 – 3.47	0.031
Tumor size (cm)	1.02	0.95 - 1.10	0.527			
Sorafenib	0.89	0.51 – 1.56	0.688			
ALBI			0.769			
ALBI 1	1					
ALBI 2	1.24	0.69 – 2.24	0.468			
ALBI 3	1.16	0.46 – 2.94	0.761			
viral vs. non-viral etiology	1.03	0.60 – 1.76	0.923			
segmental portal vein thrombosis	2.10	1.04 – 4.24	0.038			
AFP > 400 ng/ml	1.16	0.68 - 2.00	0.582			
ECOG						
0	1					
1	0.91	0.43 – 1.95				
2	2.60	0.91 7.48				
multiple metastases	0.99	0.48 – 2.04	0.981			

Additional sorafenib treatment in patients with metastatic HCC treated with TACE did not result in an independent positive prognostic effect. BCLC was not entered in the Cox model as all patients were classified as BLCL C who were treated with TACE or TACE and sorafenib

*Abbreviations:* ^*1*^ *HR* hazard ratio, ^*2*^ *95% CI* 95% confidence interval

## Discussion

HCC is often diagnosed in an advanced stage with or without extrahepatic metastases. In these patients, curative treatment options are not available. According to the BCLC classification these patients are classified as BCLC stage C and sorafenib is recommended as the treatment of choice [5, 11, 20]. However, sorafenib treatment only leads to a modest improvement of OS of approximately 3 months [11]highlighting that new treatment approaches in patients with advanced HCC either with or without extrahepatic metastases are urgently needed. It has to be considered that 66-89% of advanced HCC patients do not die from extrahepatic metastatic disease but rather form intrahepatic HCC progression or cancer associated liver failure [10, 16]. These data provide the rationale for local intrahepatic treatment of HCC e.g. using TACE with the goal to improve OS due to delayed intrahepatic tumor progression. Therefore, we set out to analyze the effect of TACE on OS in patients with metastatic HCC.

47.4% of the included patients did not receive any HCC specific treatment. Apart from no available effective treatment option before 2007, the most important reason for withholding HCC treatmentwas the presence of impaired liver function. Multivariate Cox regression models revealed that liver function as indicated by the ALBI score, is an important predictor of OS in patients with metastatic HCC. These results support the importance of preserving liver function during HCC specific treatment. Retrospective assessment of the Child score was often difficult due to inaccurate assessment of the highly subjective parameters ascites and hepatic encehalopathy. Being aware of this selection bias, we decided to use the ALBI grade for measurement of liver function as it has shown good prognostic effects in patients with liver cirrhosis and HCC [19, 21, 22]. Another important reason for choosing the ALBI grade for measurement of liver function was that the ALBI grade showed a better discriminatory capacity compared to the Child score in the estimation of the prognosis in our patient cohort (by means of Harrell’s concordance index (0.64 vs. 0.57)).

As there had been no effective systemic chemotherapeutic therapy available for patients with advanced HCC before sorafenib, patients with metastatic disease were treated with TACE on an individual basis. As previous studies showed good evidence that prognosis of metastatic patients is mainly determined by intrahepatic HCC [13, 14], selected patients were treated with TACE even after sorafenib had been introduced. In many Asian centers, TACE is regularly performed in these patients even though there are no randomized controlled studies supporting this strategy [23]. Zhao et al. reported a meta-analysis of patients with advanced HCC who were treated with TACE showing a median OS of 14.0 months in the TACE group and 9.7 months in the sorafenib group [15]. This meta analysis primarily included patients with vascular invasion and in only four studies patients with extrahepatic metastases had been analyzed and these results may not reflect the efficacy of TACE in metastatic patients. These inclusion criteria may explain why OS in our patients treated with TACE was lower with 9 months. Compared to systemic treatment with sorafenib, TACE was associated with better OS in patients with metastatic HCC. There might be a bias in our patient cohort due to inclusion of patients before sorafenib. Stratifying patients according to the time point of study inclusion (before and after introduction of sorafenib), the sub-group analyses showed that in both cohorts TACE was an independent prognostic factor. These results may be the rationale for focusing on intrahepatic treatment rather than on systemic treatment in patients with metastatic HCC.

Moreover, we set out to analyze if addition of sorafenib to TACE may result in better OS. In the recently published SPACE trial, addition of sorafenib to TACE did not result in a clinically relevant improved time to progression and OS in patients with intermediate HCC [24]. After adjusting for other important parameters in multivariate Cox regression models, the combined use of sorafenib and TACE had also no additional prognostic effect in our study. It has to be considered that only in 22.7% of our patients the recommended dose of 800 mg sorafenib daily has been administered. Further, treatment duration with sorafenib was short. Clearly, these factors may have limited the efficacy of sorafenib. However, reduced doses of sorafenib and short treatment durations somewhat reflect clinical reality with this drug [25].

Only few patients had been treated with DEB-TACE while most of our patients received cTACE. It is well known that in patients with cTACE higher systemic levels of the injected chemotherapeutic agent are observed compared to patients treated with DEB-TACE which may also have a therapeutic impact on extrahepatic metastases [26]. Therefore, further studies are warranted to eluciate this important question.

Taken together, intrahepatic tumor treatment with TACE was associated with a better OS compared to treatment with sorafenib and that the combination of bothwas no independet prognostic factor. Therefore,we suggestevaluatingthe technical feasibility of TACE in all metastatic patients. However, as many patients had been treated with several sessions of TACE before development of metastases, the transarterial approach may be difficult and even not possible. In this settingstereotactic body radiation therapy (SBRT) has emerged as another innovative treatment possibility in patients with advanced HCC achieving local intrahepatic tumor control and first small studies have shown promising results [27–31]. Studies focusing on SBRT in metastatic HCC are urgently needed. Further, transarterial radioembolization with Yttrium-90 has shown good local tumor control rates compared to conventional transarterial chemoembolisation [32]. Therefore, this may be another possibility to achieve local intrahepatic tumor control in metastatic HCC and should be investigated in further prospective studies.

Noteworthy, our study has several limitations. The decision for treatment with TACE depended on many different parameters including intrahepatic tumor expansion, portal vein thrombosis, the performance status of the patients, liver function and also on the extent of the metastatic disease (patients with multiple metastases have rarely been treated). Considering these factors, retrospective analyses of OS according to different treatment approaches, are always associated with a significant detection bias and will result in overestimation of treatment efficacy. Due to the differences in baseline characteristics we performed multivariate Cox regression model to adjust for these possible confounders. Moreover, we used OS as the primary endpoint which includes cancer-related, liver-related deaths as well as other deaths. It may be important to link OS to the different causes of death, but due to the retrospective design of our study, we were not able to perform these analyses. For our multivariate model, we only had 65 cases and due to the small sample size, this analysis may be biased and should be repeated with more patients in prospective, randomized studies. Further, we did not assess intrahepatic tumor response after TACE as imaging follow-up was only available in 39 patients.

## Conclusion

Taken together, our data suggest that treatment with TACE in metastatic HCC patients with preserved liver function may be associated with better OS. Although these preliminary data have been derived from retrospective analyses, they may help to design prospective, randomized controlled trials to assess the efficacy of TACE in patients with metastatic HCC.

## Additional file

Additional file 1: Figure S1. In patients who did not receive HCC-specific treatment, impaired liver function was the main reason for the decision not to treat these patients (A: before introduction of sorafenib in dialy clinical practice, B: after introduction of sorafenib). (TIFF 64 kb)

## Abbreviations

95% CI: 95% confidence interval
AFP: Alpha-fetoprotein
BCLC: Barcelona Clinic Liver Classification
CT: Computerized tomography
HBV: Hepatitis B virus
HCC: Hepatocellular carcinoma
INR: International normalized ratio
MRI: Magnetic resonance imaging
NASH: Non-alcoholic steatohepatitis
OS: Overall survival
SBRT: Stereotactic body radiation therapy
TACE: Transarterial chemoembolisation

## References

[1] Hassan MM, Abdel-Wahab R, Kaseb A (2015). Obesity early in adulthood increases risk but does not affect outcomes of Hepatocellular carcinoma. Gastroenterology.

[2] Mittal S, El-Serag HB (2013). Epidemiology of hepatocellular carcinoma: consider the population. J Clin Gastroenterol.

[3] El-Serag HB (2011). Hepatocellular carcinoma. N Engl J Med.

[4] Hu H, Duan Z, Long X (2014). Sorafenib combined with transarterial chemoembolization versus transarterial chemoembolization alone for advanced-stage hepatocellular carcinoma: a propensity score matching study. PLoS One.

[5] Forner A, Llovet JM, Bruix J (2012). Hepatocellular carcinoma. Lancet.

[6] Llovet JM, Di Bisceglie AM, Bruix J (2008). Design and endpoints of clinical trials in hepatocellular carcinoma. J Natl Cancer Inst.

[7] Katyal S, Oliver JH, Peterson MS, Ferris JV, Carr BS, Baron RL (2000). Extrahepatic metastases of hepatocellular carcinoma. Radiology.

[8] Shuto T, Hirohashi K, Kubo S (2001). Treatment of adrenal metastases after hepatic resection of a hepatocellular carcinoma. Dig Surg.

[9] Si MS, Amersi F, Golish SR (2003). Prevalence of metastases in hepatocellular carcinoma: risk factors and impact on survival. Am Surg.

[10] Uka K, Aikata H, Takaki S (2007). Clinical features and prognosis of patients with extrahepatic metastases from hepatocellular carcinoma. World J Gastroenterol.

[11] Llovet JM, Ricci S, Mazzaferro V (2008). Sorafenib in advanced hepatocellular carcinoma. N Engl J Med.

[12] Kalyan A, Nimeiri H, Kulik L (2015). Systemic therapy of hepatocellular carcinoma: current and promising. Clin Liver Dis.

[13] Kanda M, Tateishi R, Yoshida H (2008). Extrahepatic metastasis of hepatocellular carcinoma: incidence and risk factors. Liver Int.

[14] Natsuizaka M, Omura T, Akaike T (2005). Clinical features of hepatocellular carcinoma with extrahepatic metastases. J Gastroenterol Hepatol.

[15] Zhao Y, Cai G, Zhou L (2013). Transarterial chemoembolization in hepatocellular carcinoma with vascular invasion or extrahepatic metastasis: a systematic review. Asia Pac J Clin Oncol.

[16] Okusaka T, Okada S, Ishii H (1997). Prognosis of hepatocellular carcinoma patients with extrahepatic metastases. Hepato-Gastroenterology.

[17] Bruix J, Sherman M (2011). Management of hepatocellular carcinoma: an update. Hepatology.

[18] European Association For The Study Of The Liver, European Organisation For Research And Treatment Of Cancer. EASL-EORTC clinical practice guidelines: management of hepatocellular carcinoma. J Hepatol. 2012;56(4):908–43. 10.1016/j.jhep.2011.12.001 22424438

[19] Johnson PJ, Berhane S, Kagebayashi C (2015). Assessment of liver function in patients with hepatocellular carcinoma: a new evidence-based approach-the ALBI grade. J Clin Oncol.

[20] Forner A, Gilabert M, Bruix J, Raoul JL (2014). Treatment of intermediate-stage hepatocellular carcinoma. Nat Rev Clin Oncol.

[21] Pinato DJ, Sharma R, Allara E (2017). The ALBI grade provides objective hepatic reserve estimation across each BCLC stage of hepatocellular carcinoma. J Hepatol.

[22] Pinato DJ, Yen C, Bettinger D (2017). The albumin-bilirubin grade improves hepatic reserve estimation post-sorafenib failure: implications for drug development. Aliment Pharmacol Ther.

[23] Omata M, Lesmana LA, Tateishi R (2010). Asian Pacific Association for the Study of the liver consensus recommendations on hepatocellular carcinoma. Hepatol Int.

[24] Lencioni R, Llovet JM, Han G (2016). Sorafenib or placebo plus TACE with doxorubicin-eluting beads for intermediate stage HCC: the SPACE trial. J Hepatol.

[25] Al-Rajabi R, Patel S, Ketchum NS (2015). Comparative dosing and efficacy of sorafenib in hepatocellular cancer patients with varying liver dysfunction. J Gastrointest Oncol.

[26] Varela M, Real MI, Burrel M (2007). Chemoembolization of hepatocellular carcinoma with drug eluting beads: efficacy and doxorubicin pharmacokinetics. J Hepatol.

[27] Su TS, Liang P, Lu HZ (2016). Stereotactic body radiation therapy for small primary or recurrent hepatocellular carcinoma in 132 Chinese patients. J Surg Oncol.

[28] Wang PM, Chung NN, Hsu WC, Chang FL, Jang CJ, Scorsetti M (2015). Stereotactic body radiation therapy in hepatocellular carcinoma: optimal treatment strategies based on liver segmentation and functional hepatic reserve. Rep Pract Oncol Radiother.

[29] Meng M, Wang H, Zeng X (2015). Stereotactic body radiation therapy: a novel treatment modality for inoperable hepatocellular carcinoma. Drug Discov Ther.

[30] Feng M, Brunner TB, Ben-Josef E (2015). Stereotactic body radiation therapy for liver cancer: effective therapy with minimal impact on quality of life. Int J Radiat Oncol Biol Phys.

[31] Klein J, Dawson LA, Jiang H (2015). Prospective longitudinal assessment of quality of life for liver cancer patients treated with stereotactic body radiation therapy. Int J Radiat Oncol Biol Phys.

[32] Padia SA, Johnson GE, Horton KJ (2017). Segmental yttrium-90 Radioembolization versus segmental Chemoembolization for localized Hepatocellular carcinoma: results of a single-center, retrospective, propensity score-matched study. J Vasc Interv Radiol.

